# Histone deacetylase 2 (HDAC2) attenuates lipopolysaccharide (LPS)-induced inflammation by regulating PAI-1 expression

**DOI:** 10.1186/s12950-018-0179-6

**Published:** 2018-01-10

**Authors:** Wen-Feng Fang, Yu-Mu Chen, Chiung-Yu Lin, Hui-Lin Huang, Hua Yeh, Ya-Ting Chang, Kuo-Tung Huang, Meng-Chih Lin

**Affiliations:** 1 Division of Pulmonary and Critical Care Medicine, Department of Internal Medicine, Kaohsiung Chang Gung Memorial Hospital, Chang Gung University College of Medicine, Kaohsiung, 833 Taiwan; 2 Department of Respiratory Therapy, Kaohsiung Chang Gung Memorial Hospital, Chang Gung University College of Medicine, 123 Ta-Pei Rd, Niao-Sung Dist, Kaohsiung, 833 Taiwan; 3 Department of Respiratory Care, Chang Gung University of Science and Technology, Chiayi, 813 Taiwan

**Keywords:** Histone deacetylase 2 (HDAC2), Plasminogen activator inhibitor (PAI), Lipopolysaccharide (LPS)

## Abstract

**Background:**

Sepsis is a life-threatening organ dysfunction caused by dysregulated host response to infection, and is primarily characterized by an uncontrolled systemic inflammatory response. In the present study, we developed an effective adjunct therapy mediated by a novel mechanism, to attenuate overt inflammation. LPS-treated macrophages were adopted as an in vitro model of endotoxin-induced inflammation during sepsis. Experiments were carried out using primary mouse peritoneal macrophages and the murine macrophage cell line RAW264.7, to elucidate the mechanisms by which HDAC2 modulates endotoxin-induced inflammation.

**Results:**

Results revealed that PAI-1, TNF, and MIP-2 expression were inhibited by theophylline, an HDAC2 enhancer, in a RAW macrophage cell line, following LPS-induced inflammation. Thus, HDAC2 plays an important role in immune defense by regulating the expression of inflammatory genes via the c-Jun/PAI-1 pathway. During LPS-induced inflammation, overexpression of HDAC2 was found to inhibit PAI-1, TNF, and MIP-2 expression. Following LPS stimulation, HDAC2 knockdown increased nuclear translocation and DNA binding of c-Jun to the PAI-1 gene promoter, thereby activating PAI-1 gene transcription. Furthermore, inhibition of PAI-1 by TM5275 alone or in combination with theophylline notably suppressed TNF and MIP-2 expression.

**Conclusion:**

HDAC2 can attenuate lipopolysaccharide-induced inflammation by regulating c-Jun and PAI-1 expression in macrophages.

## Background

Sepsis is a life-threatening organ dysfunction that is caused by a dysregulated host response to infection [1]. It is primarily characterized by uncontrolled systemic inflammatory response. Sepsis is an often fatal condition that may involve coagulopathy and impaired fibrinolysis. Currently available anti-inflammatory and anti-coagulation therapies are not effective for all patients with sepsis [2, 3]. Furthermore, sepsis-related mortality is high, but the effectiveness of treatment strategies generally remains poor and requires further research [4]. In our previous study, we investigated the Toll-like receptor-dependent inflammatory response after endotoxin exposure, which is typical of bacterial sepsis [5]. Here, we attempted to develop an effective adjunct therapy mediated by a novel mechanism to attenuate overt inflammation.

Histone deacetylases (HDACs) are a class of enzymes that remove the acetyl group from the lysine residues of histones, and play important roles in the regulation of gene expression [6]. A previous study showed that the amplified inflammatory responses in chronic obstructive pulmonary disease (COPD) are mediated by reduced HDAC activity [7]. Therapies that promote HDAC activity appear to be effective for the management of asthma and COPD [7, 8]. Overexpression of HDAC2 was shown to suppress LPS-induced TGF-alpha expression in a rat model of bronchopulmonary dysplasia [9]. However, further studies are required to determine whether HDAC agonists can effectively prevent excessive acute inflammation during sepsis. Our initial data revealed that histone deacetylase modulators can attenuate endotoxin-induced acute lung injury and inflammation in vitro [10].

In addition to the fibrinolytic pathway, the plasminogen activator system has also been demonstrated to play an important role in physiological and pathological processes [11]. Studies have indicated that urokinase plasminogen activator (uPA) and plasminogen activator inhibitor (PAI) levels are correlated with disease severity in patients with sepsis [12]. Furthermore, inflammation is often associated with increased levels of PAI-1, which regulates host inflammatory responses by promoting Toll-like receptor-4 (TLR4)-mediated macrophage activation and LPS-induced inflammation [13, 14] . Some studies have highlighted the potential of fibrinolytic agents, including small molecule inhibitors of PAI-1, for the treatment of sepsis [15]. TM5275 and other small-molecule inhibitors of PAI-1 represent a novel class of anti-inflammatory agents that can suppress macrophage migration [16].

HDACs have been implicated in fibrogenesis and are known to regulate PAI-1 expression [17]. Based on these previous findings, we aimed to elucidate the mechanisms by which HDAC2 modulates endotoxin-induced inflammation, and to investigate its function as a regulator of proinflammatory gene expression in preventing excessive inflammatory responses.

## Results

### Theophylline promotes HDAC2 activity and attenuates LPS-induced pro-inflammatory gene expression

**Fig. 1:**
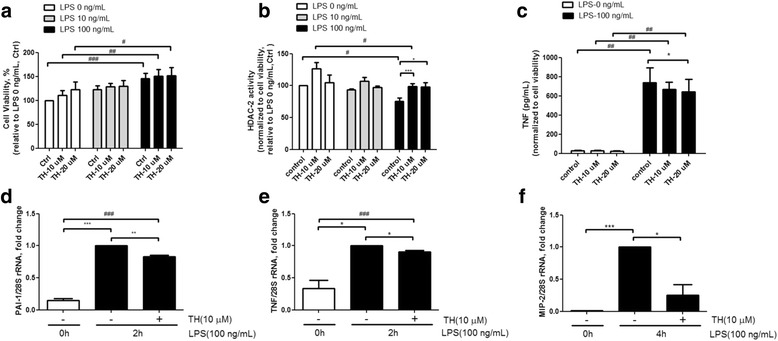
Theophylline increases HDAC2 activity and attenuates LPS-induced expression of pro-inflammatory genes. Mouse primary peritoneal macrophages cells were pretreated with theophylline (TH) for 30 min and then stimulated with LPS for 1 h. Theophylline increased cell viability (**a**), and enhanced HDAC2 activity (**b**) of peritoneal macrophages in the control and LPS-treated groups. Theophylline inhibited TNF secretion in the control and LPS-treated groups (**c**), and significantly repressed LPS-induced mRNA expression of PAI-1 (**d**), TNF (**e**), and MIP-2 (**f**) in RAW264.7 macrophages. Data are expressed as the mean relative expression ± SEM and are representative of at least three independent experiments. For all figures, * indicates *p* < 0.05; **,*p* < 0.01; ***,*p* < 0.001 in paired t test. # indicates *p* < 0.05; ##, *p* < 0.01; ###, *p* < 0.00.1 in ANOVA test

To determine the optimal pre-treatment concentration of theophylline, mice peritoneal macrophages (8 × 10^4^ cells) were seeded in 96-well plates. Cells were serum-starved for 16 h and pre-treated with 0, 10, and 20 μM theophylline for 30 min, followed by treatment with 0, 10, and 100 ng/ml LPS for 1 h or 24 h to determine the effects of theophylline on the cell viabilities of the peritoneal macrophages. Theophylline was found to slightly increase the viability of peritoneal macrophages but the observed increase was not statistically significant. For calculating the cell viability, the concentration of cytokine and the HDAC2 activity were divided by the percentage of viable cells. The cell viability of the control group was set as 100%. Treatment with theophylline for 1 h increased HDAC2 activity in the control group and LPS-treated group (Fig. 1a-b). To investigate whether treatment with an HDAC activator can influence the inflammation index, peritoneal macrophages were pre-treated with 0, 10, and 20 μM theophylline for 30 min, followed by treatment with 100 ng/ml LPS for 24 h. Culture media were collected and analyzed via TNF ELISA. Results revealed that theophylline slightly reduced TNF secretion in the LPS-treated group (Fig. 1c). In addition, theophylline significantly inhibited LPS-induced mRNA expression of PAI-1, TNF, and MIP-2 in RAW264.7 macrophages (Fig. 1d-f).

### HDAC2 overexpression attenuated LPS-induced secretion of PAI-1, TNF, and MIP-2, and enhanced uPA secretion

**Fig. 2:**
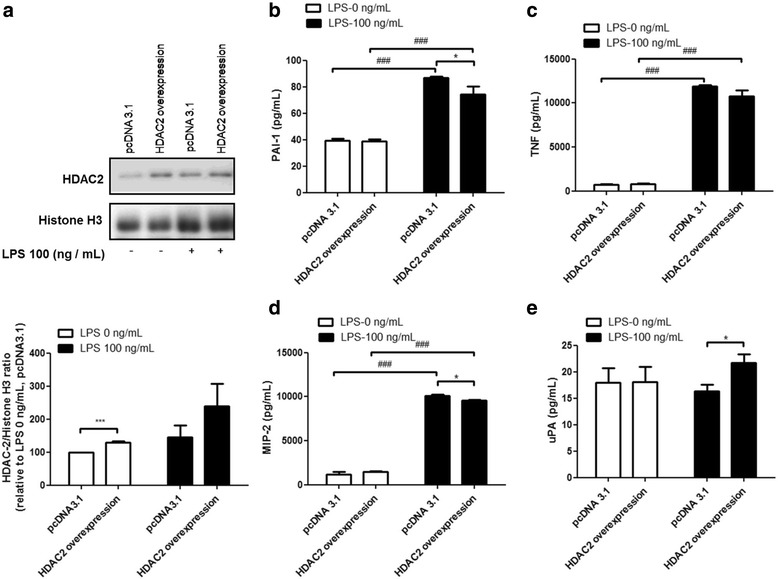
Overexpression of HDAC2 attenuates LPS-induced PAI-1, TNF, and MIP-2 secretion, but enhances uPA secretion. RAW264.7 cells were transfected with HDAC2 expression vector for 48 h, and then stimulated with 100 ng/ml LPS for 2 h. HDAC2 overexpression resulted in a 1.5-fold increase in protein levels of HDAC2 (**a**). HDAC2 inhibited LPS-induced secretion of PAI-1 (**b**), TNF (**c**), and MIP-2 (**d**), but increased uPA secretion (**e**). Data are expressed as the mean relative expression ± SEM of at least three independent experiments

To confirm the role of HDAC2 in regulating LPS-induced inflammation, we transfected RAW264.7 cells with a HDAC2 expression vector. 48 h after transfection, the RAW264.7 cells were treated with LPS (100 ng/ml) for 2 h. RAW264.7 cells transfected with HDAC2 showed around a 1.5-fold increase in HDAC2 protein expression, when compared to the cells transfected with the vector control (Fig. 2a). HDAC2 repressed LPS-induced secretion of PAI-1, TNF, and MIP-2 (Fig. 2b-d), but increased uPA secretion (Fig. 2e).

### HDAC2 knockdown significantly increased the secretion of PAI-1, TNF, and MIP-2, but inhibited uPA secretion under the LPS-treated condition

**Fig. 3:**
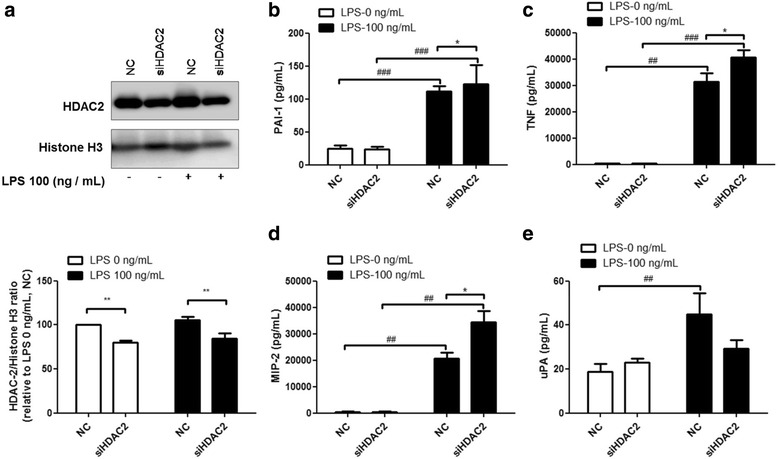
HDAC2 knockdown enhances PAI-1, TNF, and MIP-2 secretion, but represses uPA secretion during LPS-induced inflammation. RAW264.7 cells were transfected with siRNA targeting HDAC2 for 48 h, and then stimulated with 100 ng/ml LPS for 2 h. HDAC2 knockdown resulted in 20% reduction in protein levels of HDAC2 (**a**). HDAC2 significantly enhanced LPS-induced secretion of PAI-1 (**b**), TNF (**c**), and MIP-2 (**d**), but inhibited uPA secretion (**e**). Data are expressed as the mean relative expression ± SEM of at least three independent experiments

HDAC2 siRNA was transfected into RAW264.7 cells. 48 h after transfection, the RAW264.7 cells were treated with LPS (100 ng/ml) for 2 h. HDAC siRNA-transfected cells showed about a 20% downregulation of HDAC2 protein expression, relative to the negative controls (NC, scrambled siRNA) (Fig. 3a). Knockdown of HDAC2 enhanced the secretion of PAI-1, TNF, and MIP-2 under the LPS-treated condition (Fig. 3b-d), but slightly reduced uPA secretion (Fig. 3e).

### Inflammation-related transcription factors NFκB p65, c-Jun, and CEBPδ mediate the regulatory effects of HDAC2 during LPS-induced inflammation

**Fig. 4:**
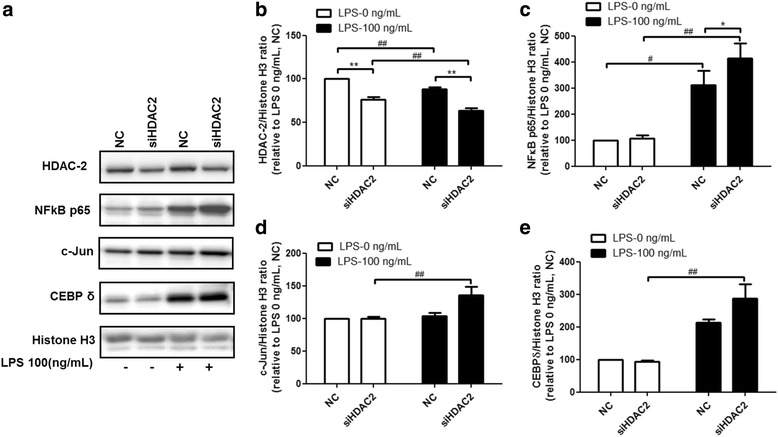
NF-κB p65, c-Jun and CEBPδ are involved in HDAC2 regulation of LPS-induced inflammation. RAW264.7 cells were transfected with siRNA targeting HDAC2 for 48 h, and then stimulated with 100 ng/ml LPS for 2 h. HDAC-2 knockdown (**a**, **b**) increased the translocation of the inflammation-related transcription factors NF-κB p65 (**a**, **c**), c-Jun (**a**, **d**), and CEBPδ (**a**, **e**) to the cell nucleus. Data are expressed as the mean relative expression ± SEM of at least three independent experiments

Previous reports have indicated that transcription factors, such as STAT1, STAT3, Nrf2, NFκB p65, c-Jun, and CEBPδ, play key roles in regulating inflammation. We utilized the UCSC Genome Browser to identify the promoter regions of PAI-1, TNF, and MIP-2. Transcription factor binding sites in the corresponding promoter regions were predicted using the PROMO and Alibaba databases. Based on the prediction results, we hypothesized that NF-κB p65, c-Jun, and CEBPδ are involved in the regulation of LPS-induced inflammation by HDAC2. HDAC2 knockdown (Fig. 4a, b) increased the nuclear translocation of NF-κB p65 (Fig. 4a,c) and c-Jun (Fig. 4a,d), and slightly promoted the nuclear translocation of CEBPδ (Fig. 4a,e).

### HDAC2 knockdown promotes the binding of acetyl-histone H3, NFκB p65, and c-Jun to the PAI-1 promoter

**Fig. 5:**
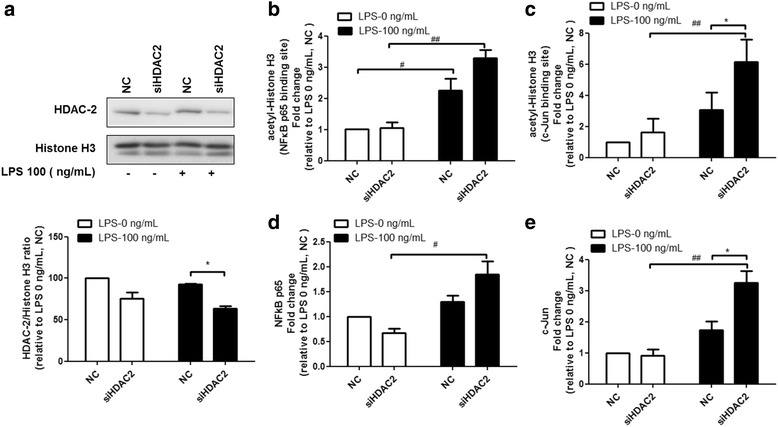
Epigenetic regulation of PAI-1 gene expression by HDAC2. RAW264.7 cells were transfected with siRNA targeting HDAC2 for 48 h, and then stimulated with 100 ng/ml LPS for 2 h. Cells were harvested and subjected to the ChIP assay to analyze the status of acetyl-Histone H3, NFκB p65, and c-Jun at the PAI-1 gene promoter. Immunoprecipitated chromatin was analyzed via qPCR using primers (shown with arrows) targeting the promoter regions of PAI-1. HDAC2 knockdown (**a**) increased the binding of acetyl-histone H3 to the NFκB p65 and c-Jun binding sites of the PAI-1 gene promoter (**b**, **c**) following LPS treatment. HDAC2 knockdown also increased the binding of NFκB p65 and c-Jun to the PAI-1 gene promoter (**d**, **e**) after LPS stimulation. Data are expressed as the mean relative expression ± SEM of at least three independent experiments

PAI-1 has been reported to play an important role in the regulation of inflammation. Previous studies indicated that PAI-1 can promote the expression of proinflammatory cytokines in bone marrow cells [13]. Based on these findings, we investigated whether HDAC2 can regulate LPS-induced TNF and MIP-2 expression via the epigenetic regulation of PAI-1. Transcription factor prediction databases predicted the presence of NFκB p65 and c-Jun binding sites at the PAI-1 promoter. Thus, we performed the ChIP assay to investigate the status of acetyl-histone, NFκB p65, and c-Jun at the PAI-1 gene promoter. Knockdown of HDAC2 (Fig. 5a) increased the binding of acetyl-histone H3 to the binding sites of NFκB p65 (Fig. 5b) and c-Jun (Fig. 5c) in the PAI-1 gene promoter after LPS treatment. HDAC2 knockdown subsequently promoted the binding of NFκB p65 (Fig. 5d) and c-Jun (Fig. 5e) to the PAI-1 gene promoter (Fig. 5d, e) after LPS stimulation, thereby inducing PAI-1 gene transcription.

### PAI-1 inhibitor TM5275, alone or in combination with theophylline, significantly inhibits LPS-induced PAI-1, TNF, and MIP-2 mRNA expression

**Fig. 6:**
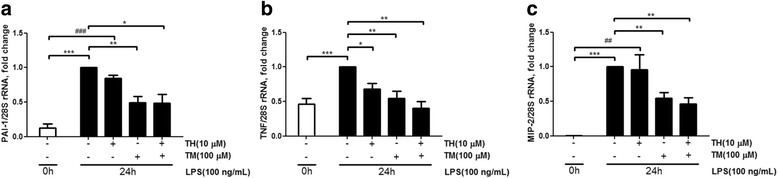
Effects of theophylline and TM5275 on RAW264.7 cells. RAW264.7 cells were pretreated with either 10 μM theophylline (TH) or 100 μM TM5275 (TM) for 30 min, and then stimulated with 100 ng/ml LPS for 24 h. Transcript levels of PAI-1, TNF, and MIP-2 were measured via qRT-PCR. Only the PAI-1 inhibitor TM5275 significantly downregulated mRNA levels of PAI-1, TNF, and MIP-2 following LPS induction. Combined treatment with theophylline and TM5275 caused a stronger inhibition of the expression of PAI-1 (**a**), TNF (**b**), and MIP-2 (**c**). Data are shown as the mean relative expression ± SEM of at least three independent experiments

Cells were treated with the PAI-1 inhibitor TM5275, to determine whether HDAC2 can repress LPS-induced TNF and MIP-2 expression through its effects on PAI-1. RAW264.7 cells (8 × 10^5^ cells) were seeded in six-well plates for 16 h and pre-treated with 10 μM theophylline or 100 μM TM5275 for 30 min, followed by treatment with 100 ng/ml LPS for 24 h. LPS treatment effectively induced mRNA expression of the proinflammatory genes PAI-1 (Fig. 6a), TNF (Fig. 6b), and MIP-2 (Fig. 6c), when compared to the cells subjected to normal culture conditions (referred to 0 h). Theophylline slightly inhibited the LPS-induced expression of PAI-1 (Fig. 6a) and MIP-2 (Fig. 6c), but significantly inhibited TNF expression (Fig. 6b). Treatment with the PAI-1 inhibitor TM5275 significantly inhibited LPS-induced mRNA expression of PAI-1 (Fig. 6a), TNF (Fig. 6b), and MIP-2 (Fig. 6c). Combined treatment with theophylline and TM5275 more effectively downregulated the expression of PAI-1 (Fig. 6a), TNF (Fig. 6b), and MIP-2 (Fig. 6c).

## Discussion

HDAC-regulated histone acetylation is an important process in epigenetic regulation. Histone acetylation leads to the unwinding of the chromatin structure, thereby allowing transcription factors to access the promoter sites, and activate gene transcription and the synthesis of inflammatory proteins [18]. Histone deacetylase 2 (HDAC2) reverses this process and switches off inflammatory gene transcription [19]. On the other hand, HDAC2 reduction activates inflammatory gene transcription and further regulates the production of inflammatory cytokines [20].

HDAC modulators have been used in various therapeutic applications aside from cancer treatment. HDACs have been studied because of their utility in the treatment of chronic inflammatory disorders [21]. Theophylline is a bronchodilator that acts as an HDAC agonist [22]. Low doses of theophylline have been used to reduce inflammation in patients with COPD. In acute disease, TNF is considered the key innate effector responsible for the rapid control of infection [23]. We measured TNF and MIP-2 levels based on previous studies [10, 24]. Our findings revealed that theophylline can partially reduce TNF secretion in murine primary peritoneal macrophages. However, the expression levels of PAI-1, TNF, and MIP-2 were evidently reduced by theophylline treatment in a RAW macrophage cell line following LPS-induced inflammation. For calculating the cell viability, the concentration of cytokine and the HDAC2 activity were divided by the percentage of viable cells. Doing so is advantageous, in that it could avoid the confounding effects of the differing LPS concentrations and other interventions that can influence cell viability. Other studies also show that LPS can influence mouse macrophage cell viability [25, 26] . In order to reduce the interference of serum cytokines, both the mice peritoneal macrophages and the RAW 264.7 cell lines were serum starved for 16 h in our study. LPS requires the LPS-binding protein, which is present in serum, to initiate its full inflammatory signaling cascade via TLR4; therefore, a lack of LPS-binding protein probably nullified an otherwise potent stimulus for TNF release. Although small changes were observed, the clinical effects could be greater if theophylline is used in combination with a steroid, as used in the asthma and COPD studies [22]. Reduction of HDAC2 expression in COPD could contribute to the insensitivity to corticosteroids [19]. Addition of theophylline (an HDAC2 activator) can increase sensitivity to steroids. Low doses of theophylline can be used in combination with a steroid, as a late-line therapy as used in the asthma and COPD studies [27, 28]. Theophylline combination therapy, which has anti-inflammatory characteristics, may be used for our future studies on sepsis.

In our study, it was noted that the effect of theophylline on LPS-induced MIP-2 secretion in RAW cells was greater than compared its effect on LPS-induced TNF and PAI-1 release. A research showed p54 expression after LPS stimulation, and that p54 inhibits LPS-induced TNF and MIP-2 expression, probably by regulation at the posttranscriptional levels [29]. The mouse proximal MIP-2 promoter contains two AP-1-like sites, one NF-kB site and one c-Jun site; these binding sites contribute to LPS-induced MIP-2 gene expression in RAW 264.7 cells [26, 30]. Especially, one study showed that HDAC modulation increased AP-1 DNA binding activity dose-dependently, when compared to another transcription factor [18]. The two AP-1 sites, which are susceptible to HDAC modulation, may provide a possible explanation about why the effect of theophylline on LPS-induced MIP-2 secretion is greater. As the time course studies of cytokine expression show a peak of MIP-2 at the 4-h time period, MIP-2 levels after 4 h were checked [31]. Stimulation with LPS for longer time periods (4 h for MIP-2 and 2 h for other transcription factors) may amplify the difference.

To elucidate the role of HDAC2 in acute inflammation, we performed an HDAC2 plasmid or siRNA transfection of the mouse macrophage cell line RAW264.7. Due to restrictions like the difficulty in acquiring primary peritoneal macrophages, and the consideration for animal welfare, transfections involving HDAC2 knockdown and overexpression were only performed in RAW cells. Results showed that HDAC2 can act as a potent and selective negative regulator of proinflammatory gene expression, and prevent excessive inflammatory responses. Differences between RAW and primary cell data highlight the limitations of the current study. Besides, although primary peritoneal macrophages were obtained from mice, animal studies cannot completely replicate human conditions. Further studies are needed for a better understanding of these aspects.

Macrophage activation is a critical step in the host response against bacterial infections [32]. A previous study showed that the uPA kringle domain and plasminogen activator inhibitor-1 (PAI-1) can enhance LPS-induced inflammatory cell responses through JNK-mediated pathways [33]. In addition, PAI-1 has been demonstrated to participate in inflammation and tissue remolding in various diseases. Furthermore, serum PAI-1 levels were found to be associated with systemic inflammation in COPD [34]. C/EBP is enriched at the −209/−200 region of the PAI-1 promoter in RAW cells [35]. Multiple studies have also demonstrated a direct relationship between TNF and PAI-1 expression [36–38]. PAI-1 induced an upregulation of the proinflammatory cytokines transcripts TNF and MIP-2 in macrophages [13]. Studies have demonstrated that a PAI-1 inhibitor (TM5275) blocks the binding site between PAI-1 and tPA [39], and decreases active PAI-1 levels in mice [40] and in human cells [41]. According to our results, the PAI-1 inhibitor reduced the expression levels of TNF and MIP-2. Interestingly, treatment with the PAI-1 inhibitors also effectively reduced PAI-1 mRNA, and this was consistent with the results from previous studies [16, 42]. Although the mechanism was not clear, the results suggest the presence of a positive feedback loop between the activity and expression of PAI-1 [42]. We did not investigate whether there is a time dependent effect of PAI-1 inhibition on the suppression of LPS-induced PAI-1 mRNA expression. However, the transcript expression levels of TNF and MIP-2 induced by PAI-1 is time-dependent, dose-dependent, and related to the role of the functional sites of PAI-1 [13]. Consistent with earlier findings, our results suggested that PAI-1 is involved in the early sepsis process [43]. Further studies are needed to explore the details of the inhibition of PAI-1 alone or in combination with an HDAC2 activator like theophylline, for clinical use.

In fig. 2, we found that up-regulation of uPA secretion was associated with the LPS-induced suppression of TNF, PAI-1 and MIP-2. uPA is under the tight control of its inhibitor, PAI-1 [44]. PAI-1 inhibits urokinase-type plasminogen activator (uPA) by binding to its active form, leading to the attenuation of plasminogen activation [45]. In this study, we demonstrated that overexpression of HDAC2 repressed PAI-1, TNF, and MIP-2 secretion after LPS stimulation. On the other hand, HDAC2 knockdown increased nuclear recruitment and DNA binding of the transcription factors c-Jun (NF-kB p65 to lesser extent) at the PAI-1 gene promoter, thereby activating PAI-1 gene transcription during LPS-induced inflammation. In addition, inhibition of HDAC activity could down-regulate the expression of urokinase plasminogen activator [46]. Conversely, HDAC overexpression may partially activate uPA expression. PAI-1, a uPA inhibitor, was suppressed by HDAC2, resulting in the upregulation of uPA expression. TM5275, a small-molecule PAI-1 inhibitor, alone or in combination with theophylline, effectively inhibited the secretion of PAI-1, TNF, and MIP-2 in LPS-induced inflammation, suggesting that HDAC2 regulates LPS-induced inflammation via PAI-1.

In Figs. 2, 3, and 4, we did not show data for different levels of HDAC2 knockdown. There is a study showing that mRNA levels of TNF decreased in a dose-dependent manner due to HDAC modulation, when compared to the case with LPS-stimulated cells [18]. Other studies have demonstrated that HDAC2 knockdown can dose- dependently activate NF-κB signaling, and induce TNF expression in macrophages [47, 48].Therefore, we suggest that there is a correlation between the levels of HDAC2 knockdown or overexpression, and the effects of their functions on LPS-induced analyte release or nuclear transcription factor levels. However, this is out of the scope of the present study.

We used LPS-induced inflammation in macrophages as an in vitro model of sepsis. Sepsis is a life-threatening condition, and is one of the leading causes of mortality worldwide; patients’ outcomes are dependent on their immune response [49, 50]. Despite the efforts of medical professionals, antibiotic administration within 1 h from the time of diagnosis can be difficult [51]. In the clinical setting, attenuating overt inflammation is critical for the treatment of endotoxin (LPS)-induced sepsis. The use of adjuvant drugs that act as selective negative regulators is a promising approach to prevent amplified inflammatory responses. Theophylline is a well-established bronchodilator that acts as an HDAC activator, and is therefore a good candidate drug for reducing inflammation. However, further studies are required to validate the use of theophylline for the treatment of sepsis. Future studies should investigate epigenetic heterogeneity and explore strategies to avoid potentially harmful effects of candidate drugs such as theophylline [52].

We demonstrate that the weak HDAC2 activator theophylline, and HDAC2 knockdown and overexpression affect the LPS-induced expression of PAI-1, TNF and MIP-2, to different degrees. This was associated with changes in Acetyl Histone H3 enrichment at the PAI-1 and TNF promoter sites, and the enhanced recruitment of p65 and c-Jun to the native PAI-1 promoter. The role of PAI-1 is worth understanding, and necessitates further study.

## Conclusions

Overexpression of HDAC2 was found to significantly reduce PAI-1, TNF, and MIP-2 secretion during LPS-induced inflammation. HDAC2 knockdown promoted nuclear translocation, and the binding of the c-Jun transcription factors to the PAI-1 gene promoter, thereby activating PAI-1 gene transcription. Furthermore, inhibition of PAI-1 by TM5275 alone or in combination with theophylline downregulated TNF and MIP-2 expression. Our findings suggested that HDAC2 plays an important role in regulating the expression of inflammatory genes during LPS-induced inflammation. The c-Jun/PAI-1 pathway is a key regulator of the immune response. HDAC2 attenuates lipopolysaccharide (LPS)-induced inflammation by regulating PAI-1 expression.

## Methods

### Animals

Wild-type (C57BL/6 J) mice were obtained from the National Laboratory Animal Center (Taiwan). Procedures for the care of the animals and the experiments were followed in accordance with the Guide of the Care and Use of Laboratory Animals from Chang Gung Memorial Hospital institutional animal care and use committee. The Institutional Biosafety Committee of Chang Gung University approved the experimental procedures.

### Primary peritoneal macrophage studies

To induce the accumulation of macrophages in the peritoneal cavity, mice (C57BL/6 J) were intraperitoneally injected with 1.5 ml of 4% thioglycollate broth (Sigma-Aldrich, USA) as previously described [31]. Three days after thioglycollate injection, macrophages were recovered by peritoneal lavage with HBSS (Gibco, USA). The recovered cells were washed twice in RPMI 1640 (Gibco, USA).

### Primary peritoneal macrophage culture and reagents

Primary peritoneal macrophages were grown in RPMI 1640 medium containing 10% fetal bovine serum (FBS) (Gibco, USA). Primary peritoneal macrophages were seeded in cell culture plates (NUNC, Denmark) and serum starved for 16 h. Cells were pre-treated with theophylline (Tocris, Bristol, UK), and subsequently treated with LPS (Sigma-Aldrich, USA) for determination of cell viability, HDAC2 activity, and TNF expression.

### Cell viability assessment

Mouse peritoneal macrophages (8 × 10^4^) were seeded in a 96-well plate and serum-starved for 16 h. To determine the effects of theophylline and LPS on the cell viabilities of peritoneal macrophages, cells were pre-treated with 0, 10, and 20 μM theophylline (Sigma-Aldrich, USA) for 30 min, and subsequently treated with 0, 10, and 100 ng/ml LPS (Sigma-Aldrich, USA) for 1 h to 24 h. Cell viabilities at different treatment conditions were determined using a CellTiter-Glo® Luminescent Cell Viability Assay kit, according to the manufacturer’s instructions (Promega Corporation, Madison, USA).

### HDAC2 activity analysis

Mouse peritoneal macrophages (1 × 10^5^ cells) were seeded in a black 96-well plate (Griner, Austria). HDAC2 activity at the different treatment conditions was evaluated using an HDAC-Glo™ 2 Assay kit, according to the manufacturer’s instructions (Promega Corporation, Madison, USA).

### RAW264.7 cell culture and reagents

The murine macrophage cell line RAW264.7 was purchased from Food Industry Research and Development Institute, Taiwan. RAW264.7 cells were grown in Dulbecco’s modified Eagle’s medium (DMEM, Gibco, USA) containing 4 mM L-glutamine (Gibco, USA); it was adjusted to contain 3.7 g of sodium bicarbonate (Sigma-Aldrich, USA) and 10% FBS (Gibco, USA).

### Cytokine analysis

Mouse peritoneal macrophages or RAW264.7 cells (8 × 10^5^ cells) were seeded in culture plates. Cytokine levels under the different treatment conditions were determined via ELISA, according to the manufacturer’s instructions (PAI-1, TNF, and MIP-2: R&D Systems, USA; uPA: CUSABIO, China).

### Real-time quantitative reverse transcription PCR (qRT-PCR)

RAW264.7 cells (8 × 10^5^ cells) were seeded in culture plates. Expression levels of the target genes under the different treatment conditions were measured via real-time quantitative reverse transcription PCR. Total RNA was isolated from cells using a commercial kit (Zymo Research, USA), and then reverse-transcribed using an RT reagent Kit (TaKaRa, Japan). Primer sequences used for qPCR were as follows: PAI-1 (forward: 5’-AGGGCTTCATGCCCCACTTCTTCA-3′ and reverse: 5’-AGTAGAGGGCATTCACCAGCACCA-3′), TNF (forward: 5’-TACTGAACTTCGGGGTGATTGGTCC-3′ and reverse: 5’-CAGCCTTGTCCCTTGAAGAGAACC-3′), MIP-2 (forward: 5’-TGGGTGGGATGTAGCTAGTTCC-3′ and reverse: 5’-AGTTTGCCTTGACCCTGAAGCC-3′), and 28S rRNA (forward: 5’-GTTCACCCACTAATAGGGAACGTGA-3′ and reverse: 5’-GGATTCTGACTTAGAGGCGTTCAGT-3′). qRT-PCR was performed using a ready-to-use hot start reaction mix for SYBR Green I-based real-time PCR, on a LightCycler® 480 Instrument (Roche Molecular Systems, USA).

### Transfection of HDAC2 expression vector

HDAC2 expression vector was customized by Protech Technology Enterprise Co., LTD, Taiwan. RAW264.7 cells (8 × 10^5^ cells) were seeded in six-well plates (NUNC, Denmark) and transfected with the HDAC2 expression vector using liposomes (Lipofectamine® LTX with Plus™ Reagent, Invitrogen, USA) for 48 h, followed by treatment with 100 ng/ml LPS for 2 h. Cell pellets were subjected to western blotting to evaluate their protein expression levels. Cell culture media were collected and subjected to cytokine analysis.

### Small interfering RNA (siRNA) transfection against HDAC2

RAW264.7 cells were transfected with siRNA targeting HDAC2 (Sigma-Aldrich, USA) using liposomes (Lipofectamine® RNAiMAX Reagent, Invitrogen, MA USA), according to the manufacturer’s instructions. Cells were cultured for 48 h in complete DMEM, and subsequently treated with 100 ng/ml LPS for 2 h. The effects of gene silencing were confirmed via western blotting. Culture supernatants were subjected to cytokine analysis.

### Nuclear and cytoplasmic extraction

RAW264.7 cells were collected for the separation and preparation of cytoplasmic and nuclear extracts. Extraction was performed using the Thermo Scientific NE-PER Nuclear and Cytoplasmic Extraction Reagent, according to the manufacturer’s instructions (Thermo, Rockford, IL, USA). Extracts were stored at -86 °C until use.

### Western blotting

Western blotting was performed to measure the expression levels of nuclear HDAC2, NF-kB p65, c-Jun, and CEBPδ. The cytoplasmic and nuclear concentrations of each RAW264.7 cell sample were assayed using a Pierce BCA Protein Assay Kit (Thermo, Rockford, IL, USA), according to the manufacturer’s protocol, with BSA as standard. For western blotting, 10 μg of protein was loaded and run on a 10% Tris-HCl SDS polyacrylamide gel. Proteins were electrotransferred onto a PVDF membrane (Millipore, USA), and then blocked with 5% non-fat dry milk and 20 mM TBS containing 0.1% Tween 20. After blocking, the membrane was incubated overnight at 4 °C with rabbit polyclonal or mouse monoclonal primary antibodies against HDAC2 (Millipore, USA), NF-κB p65 (Millipore, USA), c-Jun (Proteintech, USA), CEBPδ (Rockland, USA), and histone H3 (Millipore, USA) (as nucleus internal control) at dilutions ranging from 1:1000 to 1:2000 in 2% BSA. Next, the membrane was incubated with anti-rabbit (Jackson ImmunoResearch, USA) or anti-mouse (R&D Systems, Minneapolis, MN, USA) immunoglobulin HRP-conjugated secondary antibodies at dilutions ranging from 1:10,000 to 1:40,000 in 5% non-fat dry milk. After washing five times, bands were detected using ECL western blotting detection reagents (Advansta, USA). Signals on X-ray film (Fujifilm, Japan) were scanned and analyzed using the Image J software (National Institutes of Health, USA).

### Chromatin immunoprecipitation (ChIP) assay

RAW264.7 cells were transfected with siRNA targeting HDAC2 using liposomes for 48 h, followed by stimulation with 100 ng/ml LPS for 2 h. After stimulation, cells were harvested and subjected to the ChIP assay, according to manufacturer’s instructions (Millipore, USA). Immunoprecipitated DNA was then analyzed via qPCR using primers targeting a proximal NFκB response element (forward: 5’-CAGCACTGTCAGGGTCCATA-3′ and reverse: 5’-AGAGCCTACAAAGCCTGGTG-3′) and a proximal c-Jun response element (forward: 5’-GCCTTGGTATCTGTTTACTGGA-3′ and 5′-reverse: GGGGTTCACATATTGTCATCTT-3′) in the promoter regions of PAI-1. All ChIP data are presented as fold change in the signal-to-input ratio, relative to the control.

### Statistical analysis

For each treatment condition, all samples were prepared and analyzed at the same time. Each intervention group was repeated at least thrice. Data are presented as mean ± SEM of each experimental group. Statistical differences were analyzed via paired *t*-test and ANOVA with a post hoc test where feasible. GraphPad Prism version 5.00 for Windows (GraphPad Software, La Jolla, California, USA) was used.

## Abbreviations

(ChIP): Chromatin immunoprecipitation
(HDAC2): Histone Deacetylase 2
(LPS): Lipopolysaccharide
(PAI): Plasminogen activator inhibitor
(TLR4): Toll-like receptor-4
(uPA): Urokinase plasminogen activator
